# Advances in Plasma and Laser Engineering (Second Edition)

**DOI:** 10.3390/ma19040736

**Published:** 2026-02-14

**Authors:** Mariusz Jasiński

**Affiliations:** Institute of Fluid Flow Machinery, Polish Academy of Sciences, Fiszera 14, 80-231 Gdańsk, Poland; mj@imp.gda.pl

## 1. Introduction

Plasma and laser technologies are undoubtedly among today’s important areas of science, with applications in materials science and nanotechnology. The potential of these techniques for precise interaction with matter—both at the micro- and nanoscale—has led to the creation of new structures, coatings, and functional surfaces. Advances in plasma sources (including microwave plasmas), laser sources (including the generation of ultrashort laser pulses), and integrated process diagnostics have opened up new possibilities for producing materials with controlled physicochemical properties.

This Special Issue of *Materials* entitled “Advances in Plasma and Laser Engineering (Second Edition)” collects research and review articles presenting the latest achievements in the interaction of plasma and laser radiation with materials. The papers included here cover both fundamental and applied topics—from surface modification, through deposition and sintering processes, to the intelligent control of industrial processes and the application of artificial intelligence in material diagnostics.

## 2. Overview of the Contents of the Issue

The diversity of topics in this volume reflects the interdisciplinarity of contemporary plasma and laser engineering.

- Plasma for surface modification:

The work by Nowakowska et al. [1] describes a microwave-powered plasma pencil designed for precise surface modification, combining numerical simulations with experimental verification of electromagnetic field distributions. Kusano et al. [2] present a roll-to-roll process for the deposition of SiOx coatings on PET foils using plasma generated at atmospheric pressure—a solution with great potential for the packaging and photovoltaic industries.

- Modeling and optimization of laser processes:

Song et al. [3] analyze the effect of laser parameters on the morphology and ablation mechanisms of CFRP composites using finite element simulations. Wu et al. [4] present the optimization of laser cladding processes of WC (Co, Ni) coatings using the response surface method and FEM analysis, which allows for the improvement of hardness and wear resistance.

- Machine learning and intelligent diagnostics:

In the work of Kim et al. [5], an approach based on transfer learning and multi-sensor data analysis is presented to predict the depth of the so-called “keyhole” during the laser welding of 780DP steel. The integration of AI with laser processes is currently one of the most dynamic directions of smart manufacturing processes.

- Surface Engineering and Alloy Design:

Stojanović et al. [6] review the decarburization phenomenon during the plasma nitriding of AISI 4140 steel, indicating its importance for the structure and mechanical properties. Qu et al. [7] describe the growth kinetics and exceptional hardness of the high-entropy CoCrFeNiTi alloy produced via mechanical alloying and spark plasma sintering.

- New photothermal phenomena:

Miao et al. [8] analyze the synergistic photothermal mechanisms in the so-called “rock varnish” under the influence of laser radiation, shedding new light on photoinduced conduction and the coupling of thermal phenomena in natural and technical materials.

The articles collected in this issue show how the integration of modeling, diagnostics, and intelligent control systems is transforming plasma and laser engineering from purely empirical tools into data-driven engineering and processes for predicting future research and development directions.

## 3. Possible Future Directions for Research and Development

Analysis of the above publications and those of other authors (e.g., [9,10,11,12,13,14,15,16,17,18,19,20,21,22,23,24,25]) may lead to the conclusion that the interdisciplinarity resulting from the integration of plasma and laser technologies (including new technologies) with materials science can provide opportunities for continued research and open up new directions of research and applications in areas such as the following:

- Integrated plasma–laser processes:

Combining the plasma activation phenomenon with the precision of a laser beam enables the creation of hierarchical surface structures, selective phase transformations, and enhanced layer adhesion, which may be particularly important in electronics, energy, and biomaterials.

- Artificial intelligence in process engineering:

The use of machine learning, image processing, and real-time data analysis will enable the automatic tuning of plasma and laser parameters, increasing the efficiency and repeatability of processes.

- Sustainable plasma and laser technologies:

Low-temperature, energy-efficient, and waste-free processes are becoming increasingly important, including surface activation, sterilization, and material recycling. Reducing the energy of production processes and making them more environmentally friendly seems to be an important aspect. It should be noted that plasma and laser devices have a very short startup time compared to a classic substrate heating furnace. This has already been proven to be economically beneficial.

- Nanoscale Control and Quantum Materials:

Ultrafast lasers and plasma microdischarges will enable the precise creation of quantum dots, 2D materials, and thin layers with designed properties, for example, optoelectronic ones.

- Next-generation 3D printing and plasma/laser sintering:

The integration of plasma and laser sintering with technologies of gradual deposition and bonding of subsequent material layers based on a digital model (CAD) opens a way toward the production of gradient structures with controlled parameters with the simultaneous benefit of greater design freedom and less waste. An interesting issue in this respect is the resulting mechanical metamaterials, including lightweight sandwich structures. These mechanical metamaterials are engineered structures whose extraordinary physical properties result from deliberately designed internal geometry rather than chemical composition. They exhibit characteristics unattainable in natural materials, such as a negative Poisson ratio (auxents), negative compressibility, and high vibration damping. They are used in medicine, security, and sports. For example, auxetic materials are a unique class of materials characterized by a negative Poisson ratio. Unlike conventional plastics, they become wider when stretched and thinner when compressed. Thanks to their specific, usually cellular, structure, they offer higher resistance to cracking and denting, as well as better energy absorption.
